# Advancing Active Transportation Through Mobility Justice and Centering Community

**DOI:** 10.1089/heq.2024.0087

**Published:** 2024-10-23

**Authors:** Barbara I. Baquero, Rachel Berney, Evalynn Fae T. Romano, Olivia Hicks, Robert Getch, Crystal Hall, Stephen J. Mooney, Dori Rosenberg, K. L. Shannon, Brian E. Saelens, Katherine D. Hoerster

**Affiliations:** ^1^ Department of Health Systems and Population Health, School of Public Health, University of Washington, Seattle, Washington, USA.; ^2^ College of Built Environments, Urban Design & Planning, University of Washington, Seattle, Washington, USA.; ^3^ Beacon Hill Safe Streets, Seattle, Washington, USA.; ^4^ Daniel J. Evans School of Public Policy and Governance, University of Washington, Seattle, Washington, USA.; ^5^ Department of Epidemiology, School of Public Health, University of Washington, Seattle, Washington, USA.; ^6^ Harborview Injury Prevention and Research Center, University of Washington, Seattle, Washington, USA.; ^7^ Kaiser Permanente Washington Health Research Institute, Seattle, Washington, USA.; ^8^ Seattle Neighborhood Greenways, Seattle, Washington, USA.; ^9^ Seattle Children’s Research Institute, Seattle, Washington, USA.; ^10^ Department of Pediatrics, University of Washington, Seattle, Washington, USA.; ^11^ Department of Psychiatry and Behavioral Sciences, University of Washington, Seattle, Washington, USA.; ^12^ Mental Health Service, Seattle Division, VA Puget Sound Healthcare System, Seattle, Washington, USA.

**Keywords:** mobility, health disparities, equity, active transportation, public transportation

## Abstract

**Objectives:**

We established a community–academic–policy partnership to examine mobility challenges and opportunities by centering members of a diverse South Seattle neighborhood.

**Methods:**

Three participatory research methods were used: (a) 30- to 60-min qualitative interviews with community leaders (*n* = 12) and members (*n* = 16); (b) a photovoice with youth (*n* = 10); and (c) mobility audits. We also engaged extensively in community dissemination and advocacy.

**Results:**

Four major themes emerged: experiences with the built environment; conflicting views on promoting active transportation; experiences of danger, violence, and racism while moving in the community; and pride and connections within the community. Mobility audit findings reinforced many community member messages about needed infrastructure changes. Participants consistently expressed the need for neighborhood and city-wide structural improvements to support transportation and mobility, including enhanced public transportation; better lighting, crosswalks, sidewalks, pavement, and curb cuts; and maintenance of a neighborhood mixed-use trail. Participants shared the importance of community connection while walking, rolling, or using public transit and wanted to maintain this experience.

**Conclusions:**

Collectively, findings identified ways to increase nonmotorized transportation and public transit access, safety, and resilience, centering solutions on communities of color. We disseminated and amplified community recommendations to advance mobility justice in South Seattle via a community forum, developing a website, holding meetings with local leaders, and writing through print and electronic media. A key, novel strength of our project was the addition of community organizations, community–academic partners, and government leaders from the project’s inception. Local leaders should engage in mobility justice-focused community engagement to advance equity.

## Introduction 

Fair and just access to mobility—being able to get from place to place for essential and leisure activities—is a social determinant of health.^1^ In the United States, driving personal vehicles has been the default mobility option. However, this is neither sufficient nor just, given driving cars is associated with inactivity and cardiovascular risk,^2,3^ and motor vehicle accidents are highly prevalent and preventable leading causes of death.^4,5^ Cars also pollute and fuel climate change,^6^ with dire predicted health consequences^7^ that disproportionately affect people of color, individuals with disabilities, and low-income individuals.^1,8–10^ Given the importance of mobility for economic opportunity and the high expense of personal vehicle ownership, inadequate mobility access also affects economic well-being.^11^

Active transportation—walking, rolling (e.g., cycling, scooting, using a wheelchair) by itself or in combination with public transportation—is more inclusive, economical, and healthy than personal vehicle reliance.^12–14^ Yet active transportation is a practical option only in contexts where built and social environments include supportive features such as frequent high-quality transit, sidewalks, and adequate lighting. These features are not equitably distributed, with inequities growing. Specifically, gentrification-fueled displacement from denser and transit-rich areas hinders people of color and low-income individuals from being able to use active transportation.^15^

Governments must ensure equitable access to transportation options that do not require a personal vehicle. Yet improving mobility via policy and infrastructure changes often do not include low-income communities of color and individuals with disabilities, thereby advantaging those with more resources, and possibly fueling gentrification.^16^ Changing this approach to policy and action requires authentic and thorough community engagement to ensure planning centers community needs and vision.

A community-based participatory research (CBPR) approach,^17,18^ guided by a mobility justice framework, holds promise for addressing prior gaps. CBPR emphasizes active and equal collaborations among community members and researchers, yielding sustained and long-term community-driven actions and solutions.^17,18^ Mobility justice centers Black, Indigenous, and people of color (BIPOC) communities and acknowledges historical, structural, and intersectional barriers and solutions to advance the freedom to move safely and without fear in public spaces and freedom from displacement.^15,19,20^ Combined CBPR-mobility justice frameworks have been underutilized in empirical studies^15,20^ and are also substantially underutilized by practitioners and governments as an approach for informing policies and practices. The present study sought to overcome these gaps in practice. Table 1 depicts how we combined the CBPR approach to capture the mobility justice concepts in the framework with examples of the outputs and outcomes of the study.

**Table 1.** Examples Outputs from PATHSS Demonstrating the Combine CBPR Approach and Mobility Justice Framework

CBPR approach	Mobility justice		Example outputs
Recognize community as unit of identity Build on resources of community Promote co-learning among partners Achieve research and action Emphasis relevance of community-defined problem Apply an iterative process to maintain partnership Disseminate knowledge produced by all and to all partners Require long-term commitment by all partners	**Decolonize** Honor the voices of the often overlook in mobility conversations prioritizing their power	**Decongest** Advocate for equitable access to mobility and well-being	Community organizations reached out to researchers to develop and conduct the study Photovoice with BIPOC youth Interviews with BIPOC community members and community leaders Mobility audits Community Forum Dissemination products Website o Op-edits in local newspapers o Video and infographic o Meeting with local policymakers o Manuscripts
	**Dream** Identify solutions grounded in community knowledge and expertise	**Decriminalize** Recognize that policing and mobility do not equal safety	
	**Dignity** Center safety of people navigating their community and their sense of belonging		

BIPOC, Black, Indigenous, and people of color; CBPR, community-based participatory research; PATHSS, Participatory Active Transportation for Health in South Seattle.

## Methods

We conducted the Participatory Active Transportation for Health in South Seattle (PATHSS) study using mixed-methods guided by a CBPR-mobility justice framework. The primary research question of all PATHSS study components was intentionally broad: to understand barriers and facilitators for active transportation and mobility in South Seattle. Through this design, we set out to co-create generalizable knowledge and recommendations for researchers, practitioners, and government leaders working together to advance equitable access to mobility. However, per the University of Washington Institutional Review Board of Human Subjects regulations they deemed this study as “not involving human subjects research” per federal regulations because PATHSS *primarily* focused on generating recommendations for improving mobility in a specific Seattle community.

PATHSS was conducted in an ethnically, economically, and historically diverse^21^ South Seattle neighborhood that experiences disproportionate health risks, including higher rates of high body mass index, inactivity, pollution exposure, and traffic-related injuries and fatalities.^22–24^ Concerned by disparities, the Seattle Department of Transportation (SDOT) announced in 2019 that it would spend up to $10 million to improve South Seattle’s active transportation infrastructure, including an SDOT project to enhance an existing 3-mile bicycle and walking path.^25^ In response to this government action, several community leaders from two local mobility-focused organizations (Beacon Hill Safe Streets [BHSS] and Seattle Neighborhood Greenways [SNG]) reached out to members of our academic team, wanting to conduct a community engagement project that would ensure community voice was centered as this SDOT project launched and to use this project to broadly identify barriers, facilitators, solutions, and other observations to active transportation and mobility. Through this budding partnership, we developed the PATHSS study, which sought to center and activate community input to inform community and government leaders about needed mobility changes. PATHSS established a leadership structure composed of a community–academic–policy partnership (CAPP) that mutually developed and established its priorities. In addition to interdisciplinary university faculty, the CAPP included representatives of two local mobility-focused organizations (BHSS and SNG) and government (SDOT) leaders.

This article describes (a) 30- to 60-min qualitative interviews with community leaders and members; (b) mobility audits; and (c) a photovoice study with 10 youth, the methods and findings of which are described in a separately published article.^26^ We also describe our process for disseminating findings to community members, leaders, and decision-makers.

### Community Leader and Member Qualitative Interviews

Participants were recruited through snowball sampling, outreach to community-based organizations and their listservs, social media, and flyers in community spaces. Community leaders were eligible to participate if they work or volunteer with, represent, or advocate for South Seattle communities of color. Community members were eligible to participate if they were 13 years of age or older and reported that they live, work, go to school, use services, or travel through the focal South Seattle neighborhood. We used purposive and quota sampling for community member interviews to ensure a diverse sample for age, gender, race and ethnicity, and transportation modes used.

Semi-structured interview guides focused on identifying barriers and facilitators of mobility in the neighborhood and obtaining feedback on the planned multiuse trail project. Interviews were held by phone or secure video conferencing. Interviews were audio-recorded and transcribed verbatim. Community members were paid $30 for their time.

Analysis was carried out using a rapid template qualitative analysis,^27^ a method ideally suited for pragmatically describing textual data.^28^ First, we developed an initial coding template summarizing broad “codes” based on the interview guides, mobility justice framework, findings from our parallel work with community member youth, and by reading a random sample of responses.^26^ Codes were applied independently by two project staff. Templates were subsequently updated based on emerging topics. With discrepancies or lack of clarity, discussion occurred until an agreement was reached. Once we determined that our template sufficiently identified mobility barriers and facilitators across a host of domains, we established a “final” template used to complete and finalize coding.

### Mobility Audits

We conducted nonintrusive observations of the built and social environment using mobility audits at walksheds surrounding specific neighborhood intersections. Walksheds are defined as the area around a transit station—or any central destination—that is reachable on foot for the average person. A 5-min walkshed was used around three intersections chosen because community members routinely identified them as being important for mobility and/or needing improvement. We identified a 10-min walkshed around an intersection adjacent to a light rail station because a 10-min walk is generally considered the minimum catchment area for transit station walksheds.^29^ Audits included walking and rolling counts and use of mobility support devices by people at those intersections. Audits also evaluated sidewalk and crosswalk conditions and the presence of amenities (e.g., benches, bike lanes, street trees, transit shelter, water fountains) throughout identified walksheds.

Fifteen Urban Planning and Transportation Engineering students were trained to conduct the audits. Data were collected visually and recorded using an app the audit team created using Anvil, a Python-based app builder. Intersections were graded based on the worst curb-cut or crosswalk condition present. Students followed the standard that if one curb cut or crosswalk is inaccessible or in bad condition, then the whole intersection is not accessible. The analysis sorted the sidewalks and crosswalks by the categories: poor, fair, and good.

### Youth Photovoice

As previously published, we conducted a photovoice with 10 youth of color aged 13–18 using the same inclusion criteria as the community member interviews.^26^ Photovoice is a CBPR methodology that uses photography and storytelling in consecutive focus group sessions to collectively identify needs, set priorities, and advocate for change.^30^ We conducted a thematic content analysis of verbatim transcripts. While these findings are published separately, the insights from the photovoice were merged with PATHSS interviews and audit findings.

## Results

### Participants Characteristics

Sixteen community members were interviewed (demographic characteristics: 9 Asian, 2 mixed race, 2 Black, 2 White, and 1 Hispanic; 3 genderqueer, 1 genderqueer/female, 10 females, and 2 males; ages 17–80 [mean = 38]). One reported using a wheelchair. The 12 community leaders were executive directors, staff, and organizers of nonprofit, grassroot, and volunteer-led organizations and community centers; a pastor of a faith-based community; and a high school principal. In the previously published PATHSS photovoice study, 10 BIPOC youth (<18) participated.^26^

### Community Leaders and Member Qualitative Interviews

Four major themes emerged: experiences with the built environment; conflicting views on promoting active transportation; experiences of danger, violence, and racism while transporting and moving in the community; and pride and connections in their community. Table 2 presents themes and representative quotes. Participants consistently expressed the need for neighborhood and city-wide structural improvements to support transportation and mobility, including enhanced public transportation, lighting, crosswalks, sidewalks, pavement, curb cuts, and maintenance of the multiuse trails. Participants shared the importance of community connection while walking, rolling, or using public transit and wanted to maintain this experience. These desires competed with mixed feedback regarding bike lanes and hasty transportation infrastructure changes that fail to incorporate community input.

**Table 2.** Qualitative Feedback from Community Members, Beacon Hill, Seattle, 2020–2021

Themes	Mobility barriers, facilitators, solutions, and observations	Representative participant quotes
Experiences with the built environment	Buses Free fare for everyone Extended hours that support workers who do not work 9–5 jobs Covered bus shelters with benches and timetables More routes, especially east-west Increase on-demand ride-share services that Metro provides to link people to transit	“...how can we [expand transportation], so that [it’s] actually benefiting the existing community… not pushing them out?”
	Visibility Clearly marked crosswalks and more flashing beacon crosswalks More streetlights Street design to slow cars	“If it’s really dark… you don’t feel safe there because you can’t really see where you’re going or where you’re at or who’s around you…”
	Streets and sidewalks Curb cuts everywhere Smooth sidewalks and pavement Sidewalks clear of obstruction	“The uneven pavement... we can’t afford [for] our elders to fall. That’s a hip… or a knee problem, so that’s extremely dangerous.”
	Beacon Ave. Trail Consistent maintenance and beautification More trees for shade, esthetics, and barriers from the street Smoother pavement for safety Improved seating options Planned bike project should ensure pedestrians remain priority	“I would imagine that if it was nicely paved, if it were safer, if there were lights, if there were benches that were not only six feet apart, but more frequent... it would be a lot more used and a lot more accessible.”
Conflicting views on promoting active transportation	Some participants wanted bike lanes on Beacon Ave. Trail and throughout Beacon Hill, while some did not want bike lanes Some participants wanted to keep the parking on and around Beacon Ave. Trail, while some wanted to remove the parking In comparison to adults, youth find phone applications that provide real-time arrival information for transit to be more accurate than not and do not mind if the app is incorrect	“...I feel like there’s not that many people that ride bikes on Beacon Ave. It’s a good enough amount that it’s manageable right now, but I don’t know if people don’t ride their bikes because it’s not accommodating on Beacon Hill or they just don’t ride their bikes there.”
Experiencing danger, violence, and racism	Feelings of vigilance, powerlessness, and anticipation of harm from factors such as poor streetlights and unsafe drivers Feeling vulnerable in transit with people experiencing mental health issues (e.g., visible intoxication). Would like to be able to call for mental health care and discourage police involvement Experiencing racism when navigating public spaces with family who speak languages other than English	“He wants to feel safe while going around the community ‘cause you wouldn’t want to go around somewhere if you don’t feel safe.” *“The moment Black women leave their homes we experience anxiety because we measure our safety by the capriciousness of white people and the police. I feel like when I take the train, I’m singled out to prove fare, and the presence of transit security and fare enforcement can make it feel threatening. These kinds of interactions can be unhealthy, things like raising your blood pressure for more than just that moment and potentially for hours.”*^*^a^*^
Pride and connections in their community	Views of sunsets and the city Community spaces: parks, soccer field, and community center Appreciation for local restaurants and other businesses, particularly those owned by people of color, given the impact of gentrification on the neighborhood Strong sense of community: people in the neighborhood are friendly to one another, support and look out for one another. Organizations for social justice are present in the community Importance of fighting displacement through affordable housing and programs such as rent relief	“...we sometimes run into each other and say hi… because we recognize each other from our daily walks. It’s a very welcoming community.”

^a^ A community forum breakout room participant contributed this quote, included here because it so powerfully conveys this theme that was raised frequently across interviews and the photovoice project.

### Mobility Audits

**FIG. 1.**
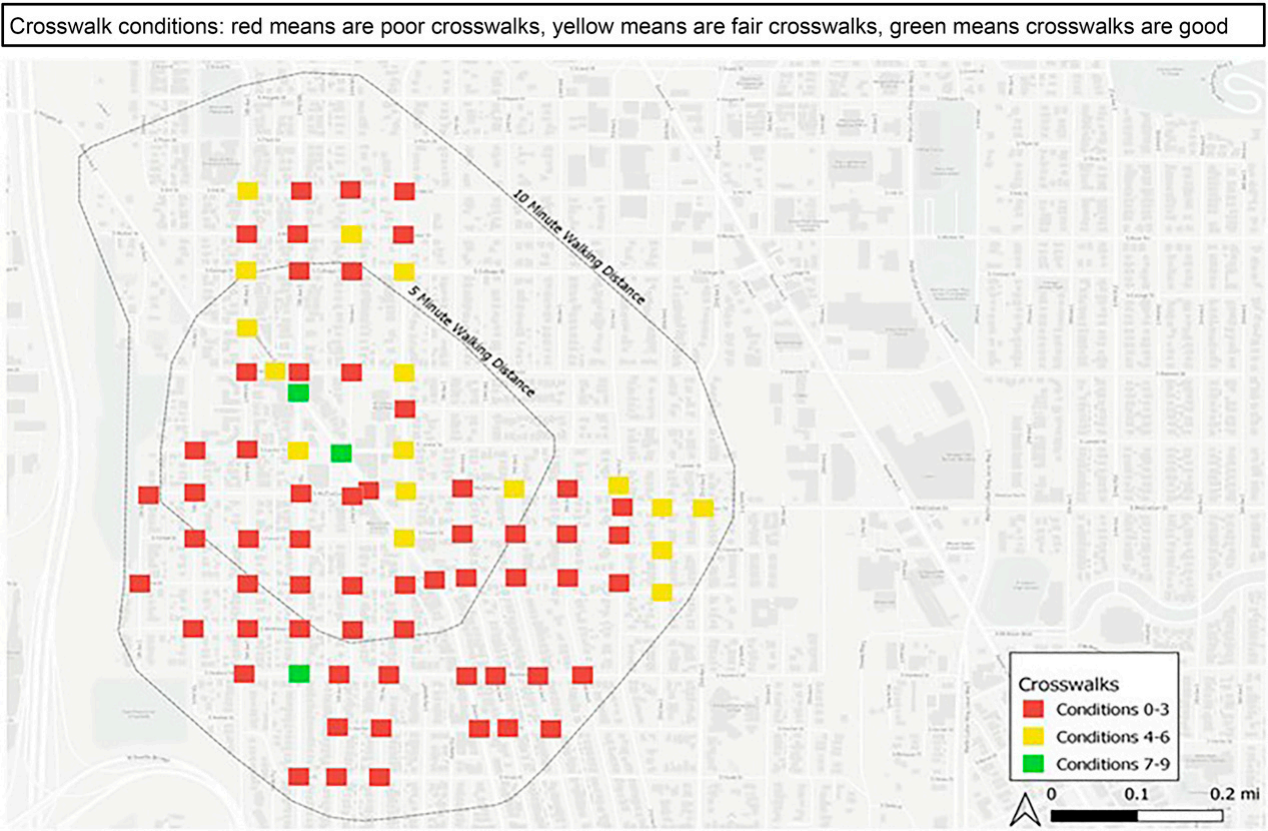
Findings from the mobility audits of key neighborhood intersections.

Mobility audit findings confirmed themes regarding infrastructure that emerged from community members. Many existing curb cuts were not designed for safe, easy wheelchair use (e.g., too narrow, obstructions). As shown in Figure 1, crosswalk and curb-cut quality varied throughout the neighborhood but was rarely good; 4% were good (conditions 7–9), 21% were fair (conditions 4–6), and 75% were poor (conditions 0–3).

Curb cuts east and west of the light rail station were generally in poor shape. Many were missing, and those that existed were too narrow for wheelchairs, did not have an accompanying curb cut on the other side of the street, and had a steep grade or no flat landing surface. Curb cuts south of the station were similar, rarely having curb cuts on all four corners of an intersection, even at busy intersections with marked crosswalks.

## Dissemination of Findings, Outreach, and Advocacy

We engaged in several activities to share key findings and advocate for corresponding infrastructure and policy changes, with our focus on dissemination led by community member encouragement: (1) a virtual community forum codeveloped with youth photovoice participants; (2) a website of the project activities and products, for example, a youth-produced short video; (3) meetings with SDOT, local, and regional transportation officials; and (4) print and online media articles.

**FIG. 2.**
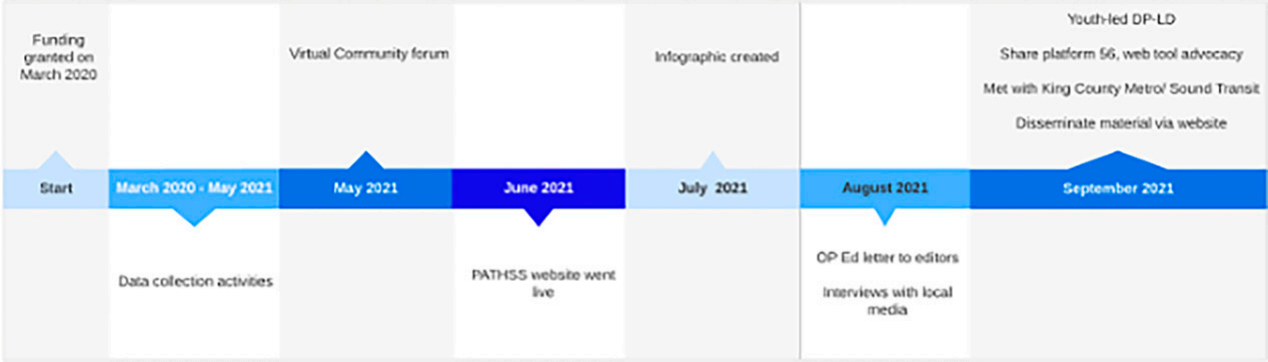
Timeline of project, events, and dissemination products.

Figure 2 depicts the timeline of events and products and a sampling of products we cocreated. Early in the project, research and BHSS partners drew on PATHSS findings to cocraft a letter to city leaders educating SDOT’s staff on the vision of the community on the proposed multiuse trail. We held a virtual community forum, coordinated by PATHSS team members and youth photovoice participants, where PATHSS findings were shared, and a youth panel discussed their experiences moving through the community. Over 70 people attended the forum, including community members, allies, transportation and city officials, and media representatives. Forum panelists and participants emphasized the important role identity plays in how they move through public spaces, the racism that drives mobility decision-making and impacts experience, and the deep costs of mobility barriers to well-being.

We developed and disseminated a website^25^ where community members can learn how to advocate for change based on PATHSS findings. From January 7, 2021, to October 3, 2023, 833 people accessed the website, with most (98%) from the United States. Among U.S. visitors, users hailed from 142 cities, with the highest number of U.S. visitors from Seattle, WA (37%), and the second highest from Cheyenne, WY (20%).

**FIG. 3.**
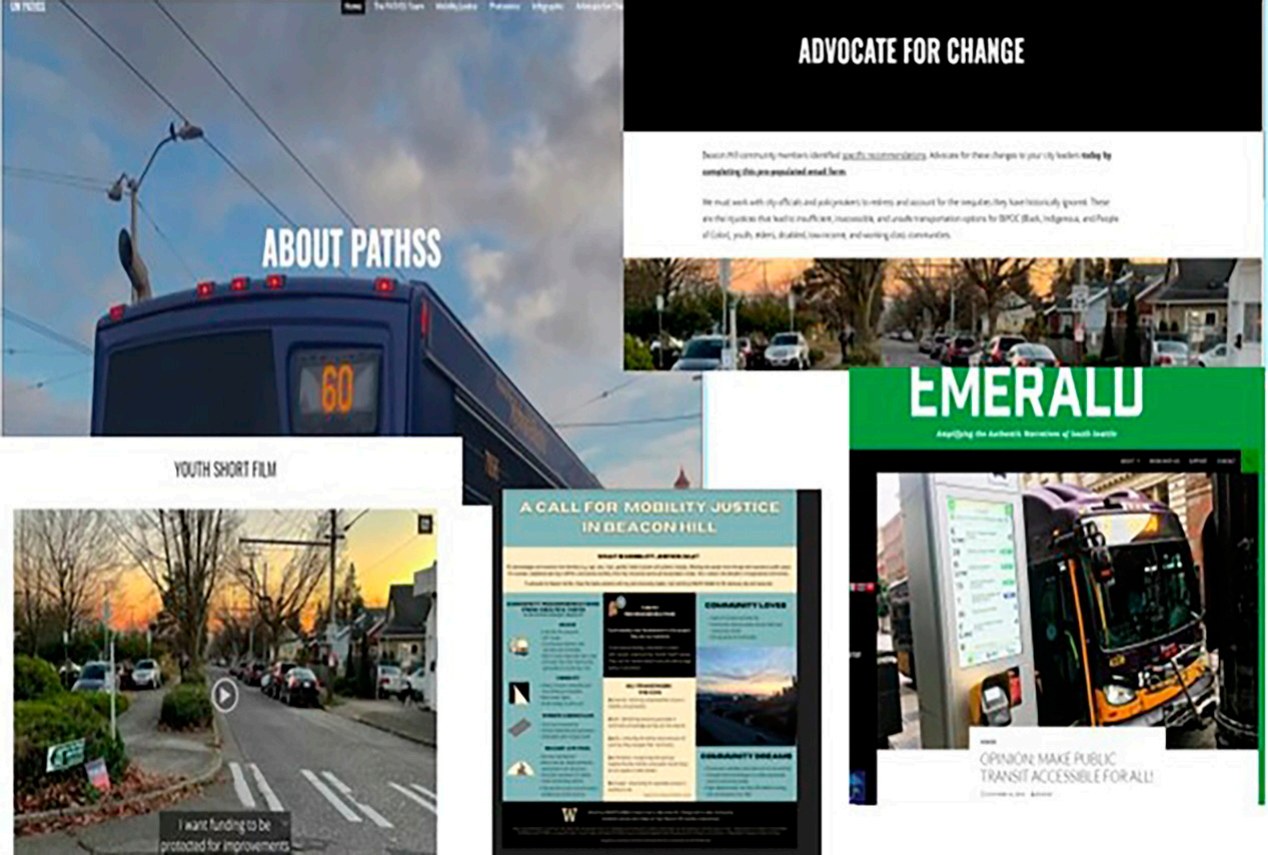
Simple tools for disseminating PATHSS key findings to advance mobility justice. PATHSS, Participatory Active Transportation for Health in South Seattle.

We developed an infographic policy brief for community members and leaders summarizing community recommendations for improved neighborhood mobility, which was broadly distributed via email to community members, leaders, and policymakers. We partnered with youth and community partners to share PATHSS findings via op-eds, letters to the editor, presentations to transportation agencies, and interviews, in local, regional, and state-wide outlets. We included all media coverage of PATHSS findings and advocacy on the website. For example, the two lead investigators cowrote an op-ed with their key community partner, calling for policy changes to reduce systemic racism-based barriers to exercise and mobility.^30^ In addition, youth photovoice participants wrote an op-ed advocating for free public transit and enhanced South Seattle mobility under the mentorship of senior PATHSS researchers. See Figure 3 for a sample of these dissemination tools.

Our community partner SNG hosted a web platform tool for community members to advocate to city leaders for key policy and infrastructure changes based on PATHSS findings. Thirty people advocated through the web tool alone. The highest use of the PATHSS website (275 users) was during this promotion campaign. Following this outreach, King County Metro and Sound Transit, the two regional public transportation agencies, met with us to discuss their policies (e.g., free transit, fare enforcement) and youth engagement. We also met with SDOT’s Vision Zero and Safe Routes to Schools leaders. PATHSS community partners (BHSS and SBG), youth, and representatives participated in these meetings and were compensated.

## Discussion

PATHSS was a CBPR study seeking to identify community mobility challenges and opportunities for change. The connection between place, transportation, and health has been studied empirically for years. However, that evidence has limitations as it often centers on individuals and how they access those places while neglecting those place’s social, economic, and historical contexts.^31^ Moreover, prior research has successfully documented transportation challenges but is too rarely paired with immediate community-driven advocacy. Conducting this project with a mobility justice lens helped focus and articulate the social action call central to CBPR, while more holistically examining people’s transportation experiences (e.g., the stress caused by experiencing harassment). Last, including government leaders in our project from its inception—even before we obtained funding—made PATHSS a unique model for ensuring community-driven policy and practice.

Infrastructure changes recommended by community members and leaders—and confirmed by the mobility audits—are evidence-based strategies for creating safe and walkable communities.^32,33^ Improving safe and fair public transit access is critical for advancing public health and equity^14^ in the context of dramatically reduced ridership and funding during the COVID-19 pandemic.^34^ Participants advocated specifically for free transit for youth or all people, implemented in Kansas City, MO, in 2019,^35,36^ and for students and low-income individuals in parts of Los Angeles, CA, in 2021.^37^ Funding for free fare systems can come from various sources, including local and federal funding and employer subsidies.^38–40^ In addition, fare collection and enforcement are costly, so eliminating those can help offset the loss of fare revenue.^41^ King County (of which Seattle is a part) made all transit free for youth 18 and younger beginning September 2022.^42^ Even with the free youth transit victory, PATHSS youth and community member participants requested additional changes that would help increase transit access, including expanded hours, more comfortable and useful shelters, and increased reliability. Transit agencies should implement these changes to further improve mobility.

As mobility justice indicates, fair, free, and safe movement in communities goes beyond physical transportation. Community members shared concerns that infrastructure improvements drive gentrification and displacement. They shared their concerns about the well-being of unhoused individuals and mentioned the importance of affordable housing. Many expressed concerns about addressing safety with police, given Black and American Indian/Alaska Native people are significantly more likely than White people to be killed by police.^43^ Government leaders must work to reduce displacement, improve access to affordable housing, tackle harms imposed by policing and criminal (in)justice systems, and dismantle the ways in which systemic racism undergirds harmful policies and practices.

## Recommendations

Based on the PATHSS process and findings, our research team has identified several recommendations for researchers, government staff and officials, and practitioners working together to advance health equity through mobility justice:

1. Researchers, government staff, and officials: maintain proactive, ongoing communication with community partners and experts, working around partners’ schedules.
2. Prioritize the voices of community partners, persons with disabilities, BIPOC, youth, and elders, who are most impacted by policy decisions, yet are often excluded from decision-making.
3. Regularly share and synthesize feedback with community partners and decision-makers, guided by timelines relevant to leaders and the community.
4. Adapt to changing community and partner needs.
5. Link community needs and resources with the appropriate governmental entity (e.g., city council and mayor, transportation agencies, parks) and audiences such as business leaders with a shared interest in community development—and potential influence.
6. Adopt an antioppression research framework, such as mobility justice or public health critical race praxis, to center the experiences of those most affected, examine contextual injustices, and address root causes of inequities.^44^
7. Engage in community-partnered *action* rather than simply observing and describing problems. We include here a quote from a community leader that we heard early on and that influenced our approach with PATHSS going forward: “Am I looking for what I don’t know? Am I shifting some focus and power away from the most privileged in my research? …an opportunity to do better and do more, instead of just an examination of the ways to get around and how these efforts are failing.”

### Limitations and Strengths

Several limitations should be considered when interpreting our findings. PATHSS took place at the start of the COVID-19 pandemic; how findings will translate to times less impacted by COVID-19 is unknown. As this study’s methods and findings are intentionally focused on a specific South Seattle neighborhood, findings may not generalize to other communities. However, we believe that it is likely the barriers and facilitators identified are shared by other diverse urban communities and should, therefore, be a focus of inquiry and action in other similar communities. Furthermore, the broad lessons learned about engaging community members to advance local community mobility apply.

## Health Equity Implications

PATHSS effectively collaborated with community and policy partners to identify and address South Seattle mobility challenges and opportunities. The CBPR-mobility justice approach allowed PATHSS to interpret findings and act from an antioppression stance. This allowed us to go beyond simply describing community members’ experiences, to directly and collaboratively address the current unjust and unfair systems that marginalize and oppress BIPOC community members. Our study serves as a road map for researchers, policymakers, implementers, and community members, shifting from applying an intra- and interpersonal approach to health promotion to one that is grounded on the dignity, freedom, safety, and well-being of communities.

Future work must continue to promote access to mobility, improve healthy behaviors and access to economic resources, reduce climate impacts, and strengthen community capacity for health and environmental advocacy. Such work will benefit from using a CBPR-mobility justice approach.

## Abbreviations Used

BHSS: Beacon Hill Safe Streets
BIPOC: Black, Indigenous, and People of Color
CAPP: Community-Academic-Policy Partnership
CBPR: Community-Based Participatory Research
PATHSS: Participatory Active Transportation for Health in South Seattle
SDOH: Social Determinants of Health
SDOT: Seattle Department of Transportation
SNG: Seattle Neighborhood Greenways
